# Preparation and Characterization of MgO-Modified Rice Straw Biochars

**DOI:** 10.3390/molecules25235730

**Published:** 2020-12-04

**Authors:** Xianxian Qin, Jixin Luo, Zhigao Liu, Yunlin Fu

**Affiliations:** 1 College of Forestry, Guangxi University, Nanning 530004, China; gxulxyqxx@sina.com; 2 College of Resources Environment and Material, Guangxi University, Nanning 530004, China; liu_zhi_gao@163.com

**Keywords:** pyrolysis, MgO-modified biochar, rice straw, biochar properties

## Abstract

Rice straw is a common agricultural waste. In order to increase the added value of rice straw and improve the performance of rice straw biochar. MgO-modified biochar (MRBC) was prepared from rice straw at different temperatures, pyrolysis time and MgCl_2_ concentrations. The microstructure, chemical and crystal structure were studied using X-ray diffraction (XRD), a Scanning Electron Microscope (SEM), Fourier transform infrared spectroscopy (FTIR), nitrogen adsorption desorption isotherms and Elementary Analysis (EA). The results showed that the pyrolysis temperature had significant influence on the structure and physicochemical property of MRBCs. MRBC-2 h has the richest microporous structure while MRBC-2 m has the richest mesoporous structure. The specific surface area (from 9.663 to 250.66 m^2^/g) and pore volume (from 0.042 to 0.158 cm^3^/g) of MRBCs increased as temperature rose from 300 to 600 °C. However, it was observed MgCl_2_ concentrations and pyrolysis time had no significant influence on pore structure of MRBCs. As pyrolysis temperature increased, pH increased and more oxygen-containing functional groups and mineral salts were formed, while MgO-modified yield, volatile matter, total content of hydrogen, oxygen, nitrogen, porosity and average pore diameter decreased. In addition, MRBCs formed at high temperature showed high C content with a low O/C and H/C ratios.

## 1. Introduction

For sustainability cause, rice straw is an ideal raw material for biochar because of its large production in China [1,2]. Until now, large amounts of rice straw are abandoned every year and they are dominantly burned. It is not only case the loss of resources, but also the production of carbon monoxide, sulfur dioxide and other gases, causing environmental pollution problems [3]. Thus, extensive studies for improving the value of rice straw have been carried out recently. The preparation of rice straw into biochar is one of the methods that has been heatedly discussed by people in recent years: it can reduce the amount of released solid particulates and smoke during the traditional process, such as composting and incineration [4]. Biochar is a carbon-rich organic substance produced by complete or partial pyrolysis of agricultural wastes, animal fertilizers and paper products under high temperature and hypoxic conditions, which generally contains more than 60% carbon [5,6]. The unique physical and chemical properties of biochar determine that it has multiple properties. Biochar has abundant surface functional groups, large specific surface area, high pH value, high cation exchange capacity and a large amount of nutrients necessary for plants [7]. Therefore, biochar can improve soil fertility, reduce soil bulk density, increase soil pH and improve soil quality and affect microbial community structure [8]. Meanwhile, biochar also increases plant photosynthesis and nutrient absorption and improves soil water retention [9]. In addition, biochar can be used as a pollutant adsorbent to repair soil contaminated by heavy metals [10]. Due to the stability and carbon-rich properties of biochar, substances such as CO_2_, N_2_O and CH_4_ can be sequestered, thus reducing the greenhouse effect [11]. In recent years, the adsorption of heavy metals in soil and pollutants in water by rice straw biochar has been widely reported [12,13]. Rice straw biochar has a large cation exchange capacity and can be used as an adsorbent for cationic pollutants [14].

Although biochar has many advantages, due to the limitations of biochar raw materials and preparation methods, biochar prepared under traditional pyrolysis has a smaller specific surface area and pore structure and fewer types of functional groups [15]. In order to solve these problems and further enhance its performance, it is necessary to use modified methods to activate the surface properties of biochar and improve its physical and chemical properties. Modified rice straw biochar has also been extensively studied in recent years. For example, MnFe_2_O_4_ modified biochar combines the advantages of biochar and MnFe_2_O_4_ and has a higher ability to immobilize Sb and Cd [16]. The specific surface area of MgO modification biochars, which were produced from woody biomasses at carbonization temperature of 500 °C, increased from 0.26–8.82 m^2^/g to 22.02–28.07 m^2^/g [17]. MgO modified biochar shows better phosphorus inorganics (Pi) adsorption capacity in saline soil, and can also increase the effective phosphorus content of the soil, thereby increasing rice yield [18]. In addition, MgCl_2_ modified biochar can be used as an excellent adsorbent for the treatment of eutrophic water [19]. Rice straw biochar prepared by microwave pyrolysis has the potential to adsorb CO_2_ [20]. Potassium-iron composite rice straw biochar has better adsorption capacity for nitrate, phosphate and ammonium ions [21]. The rice straw biochar modified by HNO_3_ and H_2_O_2_ introduces oxygen-containing acidic functional groups on the surface, and the effect of removing Cd^2+^ is better, because the deprotonation of hydroxyl and carboxyl groups provides more adsorption sites [22].

Moreover, the carbonization temperature has the most significant effect on the physical and chemical properties of biochar, which determines the yield and characteristics of biochar [23]. In previous studies, it was found that as the carbonization temperature increased, the yield of biochar and the amount of negatively charged oxygen-containing functional groups decreased, and a rich pore structure was eventually formed with the removal of hydrogen and oxygen [24]. The biochar prepared at a lower temperature has a smaller specific surface area but more oxygen-containing functional groups. According to literatures, it seems that MgO-modified rice straw derived from different temperatures were relatively less studied. For the reasons discussed above, if rice straw can be prepared into MgO-biochar, it can not only reduce its environmental pollution, but also tap its potential value. Even so, the relationship between the structure and composition of magnesium-modified straw biochar and the pyrolysis process and the properties of the resulting biochar is not clear. Thus, it is urgently needed to study the physical and chemical properties of pyrolysis at different temperatures in detail. This will also promote the production of biochar for specific application purposes. To address this knowledge gap, rice straw biochars were modified by MgO and produced at differing temperatures. Here, rice straw was used to prepare magnesium-modified biochar, and effect of different conditions on the structure and properties of MgO-modified straw biochar has been studied. The structure and properties of rice straw biochar before and after modification were compared, and compared with others reported in previously literature.

## 2. Materials and Methods

### 2.1. Preparation of Biochar and MgO-Modified Biochar

The feedstock (rice straw) of biochar was collected from the College of Agriculture, Guangxi University. All chemicals used in the study were purchased from Tianjing Chemical Works, Tianjing, China. They were all of the reagent grade and used without further purification. Rice straw was air-dried under natural conditions and then crushed by a pulverizer and sieved through 2 mm mesh. Biomass was pyrolyzed into biochar by a tube furnace. Rice straw biochar (RBC) produced by slow pyrolysis filled with nitrogen to maintain the hypoxic conditions and at a temperature of 300 °C at a heating rate of 8 °C min^−1^ for a retention time of 1 h.

A certain mass of biochar was impregnated with different concentrations (0.5 mol/L, 1 mol/L, 1.5 mol/L, 2 mol/L) of MgCl_2_ and stirred magnetically at room temperature for one hour. Then, the solution was filtered to obtain the impregnated biochar and dried in an oven at 103 °C for 5 h. Finally, 15 g of each sample after drying were weighed and put into a tube furnace and uniformly warmed up to pyrolysis temperature (300 °C, 400 °C, 500 °C, 600 °C) at a rate of 8 °C per minute and kept at that temperature for a certain time (0.5 h, 1 h, 2 h, 3 h) to obtain MgO-modified rice biochar (MRBC) [18]. From the table, it can be seen that the experimental conditions for MRBC-500, MRBC-1 m and MRBC-1 h are the same, but in order to facilitate comparison and discussion of the biochar properties under the different conditions, the samples are presented with the designations stated in the table and applied throughout the text. The nomenclatures of the samples prepared under different conditions are shown in Table 1.

### 2.2. Characterization of Biochar

The pH value was measured by pH meter (pHS-25) and the ratio of biochar to distilled water was 1:10 (*W/V*). The measurement was repeated three times per treatment. The surface morphology of biochars were revealed by a Scanning Electron Microscope after gold blasting (S-3400N, Tokyo, Japan). The specific surface area, porosity and pore volume of the prepared biochars were determined by N_2_ adsorption–desorption isotherms at −196 °C and measured by Automatic multi-station specific surface area and porosity analyzer (TriStarⅡ3020, Atlanta, GA, USA). Firstly, the samples were degassed at 300 °C for 3 h. The surface area of prepared biochars were estimated by Brunauer-Emmett-Teller method (BET) [25]. The total pore volume was estimated as the liquid volume of N_2_ at high relative pressures (P/P_0_ = 0.995). Microporous specific surface area and microporous volume were calculated by t-plot [26]. The mesopore surface area and the mesopore volume were calculated by the Barrett-Joyner-Halenda (BJH) method [27].

The yields of biochars were calculated based on a mass balance using Equation (1)

Yields (%) = M_1_/M_2_ × 100%, (1)

where M_1_ and M_2_ were the masses (g) of modified biochar and biochar dried after impregnation with magnesium chloride solution, respectively.

The sample was crushed (100 mesh) and thoroughly mixed with KBr at a ratio of 5:100. After that, the chemical groups of the biochars were recorded between 4000 and 400 cm^−1^, using a Fourier transform infrared spectrum analysis spectrometer (FTIR, Frontier, Thermo Fisher Scientific, Waltham, MA, USA). The X-ray diffraction (Rigaku, Tokyo, Japan) measurements were conducted with an X-ray diffractometer using CuKa radiation at 40 kV and 30 mA. The scanning rate was 2°/min with a scanning step of 0.2° from 5° to 65° (2θ). The elemental composition of biochars were determined by an elemental analyzer (Vario EL cube, Frankfurt, Germany).

## 3. Results and Discussion

### 3.1. pH and Yield Analysis

The effects of pyrolysis temperature, modifier, pyrolysis time and modifier concentration on the pH and yield of biochar are shown in Table 2. There was no regular effect of pyrolysis time and modifier concentration on pH, while the pH increased with increasing pyrolysis temperature. Overall, the pH of all samples after modification was higher than RBC-300. This reflected the alkaline nature due to the rich content of minerals in biochar [28]. As the pyrolysis temperature increases, the high boiling point substances in the feedstock volatilized, leading to an increase in the content of inorganic mineral components [29]. As a result, the pH value of biochar increases. The results were similar to Man Ho Parka [13]. Considering the pH of MgO-modified biochars, they may also be used as soil amendments to reduce soil acidity.

### 3.2. Nitrogen Adsorption–Desorption Curve

**Figure 1:**
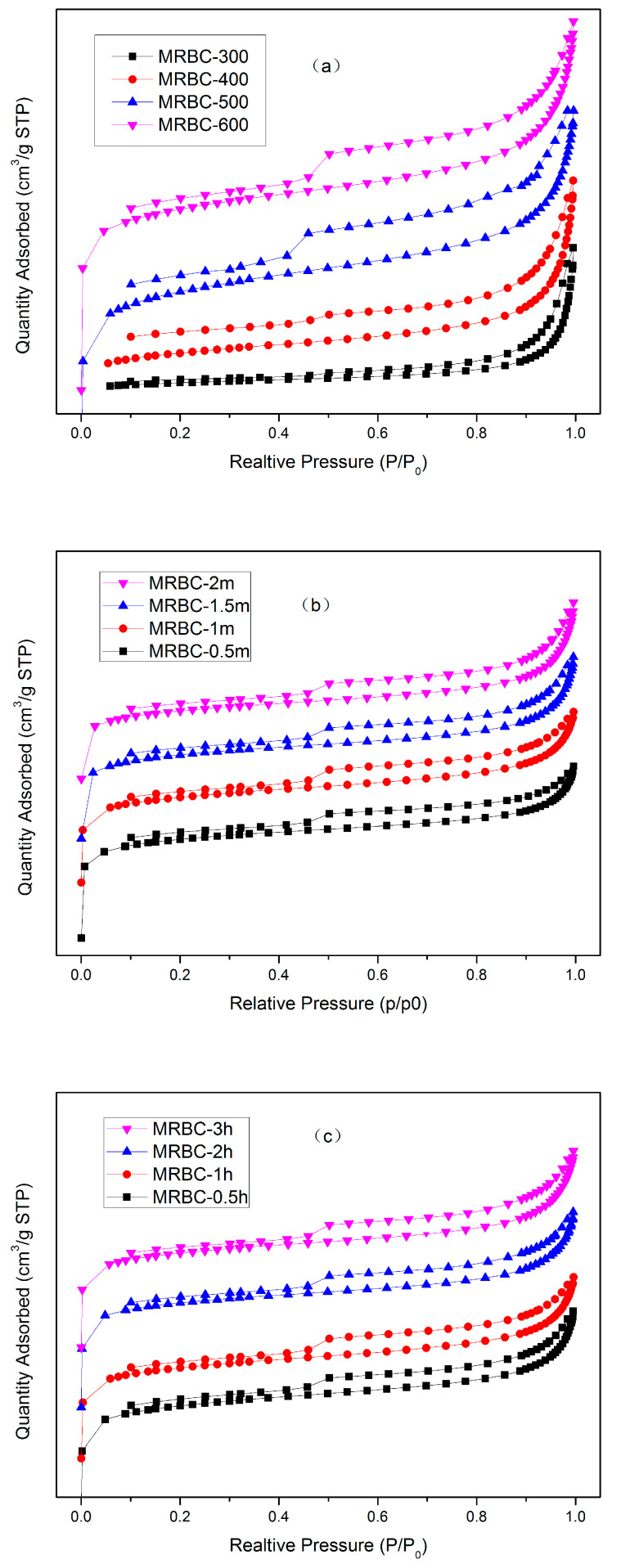
The nitrogen adsorption/desorption curves of biochar prepared at different conditions: (**a**) pyrolysis temperature; (**b**) MgCl_2_ impregnation concentration; (**c**) pyrolysis time.

The nitrogen adsorption–desorption curve onto biochar is shown in Figure 1. At a low relative pressure (≤0.01), nitrogen adsorption increases linearly with the increase of relative pressure, mainly due to the filling of micropores. However, the nitrogen adsorption and desorption curves for MRBC300 and MRBC-400 (Figure 1a) did not show nitrogen adsorption and desorption in the lower relative pressure region, mainly due to the low microporous structure of the biochar. When the relative pressure is greater than 0.1, as the relative pressure increases, the nitrogen adsorption curve shows an upwardly inclined adsorption platform. According to the IUPAC classification, it belongs to the type II adsorption isotherm, indicating that the magnesium-modified biochar has a mesopore with a large pore size. When the relative pressure is about 0.4, an approximately closed hysteresis loop is formed. It is mainly due to the strong adsorption of the mesopore, which makes the hysteresis phenomenon easy to occur due to the strong force of nitrogen and the pore during desorption. That is, the isotherm obtained during desorption does not coincide with the isotherm during adsorption. The desorption isotherm is above the adsorption isotherm and forms a hysteresis loop with the adsorption curve. Furthermore, according to the IUPAC on the phenomenon of adsorption isothermal hysteresis loop is divided into four categories. Combined with the nitrogen adsorption-desorption curve and hysteretic ring shape, the isotherm hysteresis loop of MgO-modified biochar belongs to the H4 type. The H4 hysteresis loop is mainly found in solids with slit pores, such as activated carbon, and no adsorption platform is present in the higher relative pressure region, showing adsorption limitation. This indicates that there are narrow fissure pores in MgO-modified biochar.

### 3.3. Pore Structure

Specific surface areas and pore characteristics of the MRBCs and RBC are presented in Table 3. The MgCl_2_ impregnation concentration and pyrolysis time had no significant effect on the comparative surface area, pore volume and average pore size. The maximum surface area of biochar obtained at different MgCl_2_ impregnation concentrations and pyrolysis times was 215.684 m^2^/g and 218.966 m^2^/g, respectively. The pore volume range is 0.153–0.165 cm^3^/g. The average pore size is about 3 nm.

In contrast, pyrolysis temperatures had a significant effect on the specific surface area, pore volume and average pore size of the MgO-modified biochar at a pyrolysis temperature of 1 h and an impregnation concentration of 1 mol/L. Treatment with MgCl_2_ significantly enhanced the porous structure and resulted in even higher BET surface areas and higher pore volumes than the precursor biochar. With the increase of pyrolysis temperature, the total pore volume, microporous pore volume and mesopore volume of biochar increased. The greatest change in pore volume occurred when the pyrolysis temperature was from 400 °C to 500 °C. With further increase in temperature, the specific surface area and pore structure of MRBC-500 and MRBC-600 were basically similar. It was due to the removal of volatile components from the biomass during the charring process resulting in the creation of pores, and at higher charring temperatures the condensation of tar products was reduced, which was significant for pore plugging and reduction of surface area [30]. The specific surface area of MRBCs (9.663–205.066 m^2^/g) increased significantly with the increase of the carbonization temperature below 500 °C. The surge in the specific surface area of MRBC was the result of the removal of the phenolic-OH, aliphatic alkyl and ester C=O groups linked to the aromatic nuclei, which were removed as a result of the chemical reaction caused by the carbonization temperature [31]. The specific surface area of RBC300 was very low (2.434 m^2^/g) and after modification and further heating, the volatilization of organic matter from the biochar produced a porous structure, which resulted in a much higher surface area of MRBC600 (204.579 m^2^/g). The results fell within the reported ranges of these porous parameters [32]. In comparison to AlCl_3_ modified rice straw biochar [33], the MRBC has a higher surface area (205 versus 147 m^2^/g) at 500 °C. 

Moreover, the specific surface area of MRBC-500 and MRBC-400 is superior to that of other rice straw biochar modified by H_3_PO_4_ or FeCl_3_·6H_2_O [21,34].

The average pore size of the MgO-modified biochars decreased with pyrolysis temperature as the large pores were destroyed and more small pores formed during further heating. The average pore size of the modified biochar decreased with the increase of pyrolysis temperature, but it did not change much between 500 °C and 600 °C, and the average pore size of the modified biochar was smallest at 500 °C. With the increase of temperature, the proportion of mesopores in MRBC gradually decreased from 95% to 44%, and the decrease of mesopore proportion led to the tendency of decreasing average pore diameter.

### 3.4. Pore Size Distribution

**Figure 2:**
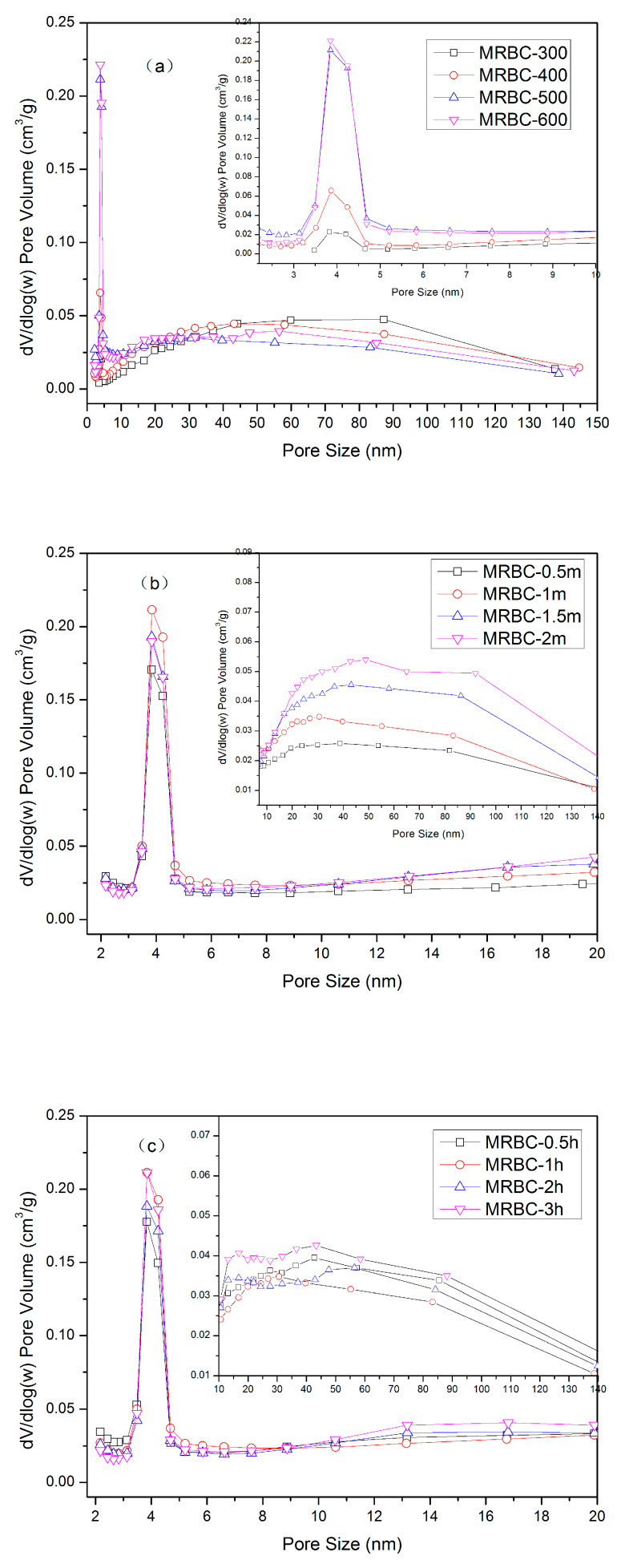
Pore size distribution of biochar prepared under different conditions: (**a**) pyrolysis temperature; (**b**) MgCl_2_ impregnation concentration; (**c**) pyrolysis time.

The pore structure distribution of MgO-modified biochars prepared at different pyrolysis temperatures, impregnation concentrations and pyrolysis times are shown in the Figure 2. MRBC-2 m has the lowest number micropores of the MgCl_2_-modified biochar at different concentrations, which was due to the blockage of excessive MgO nanoparticles acting on the pore structure of biochar [35]. The pore size distribution curves of the modified biochar at different temperatures were basically similar, but the pore size distribution in the range of 3–5 nm showed obvious growth changes with the increase of temperature. The pore structure is mainly dominated by smaller mesopores. However, the pore size distribution in the range of 30 nm to 150 nm is higher for MRBC-300 and MRBC-400 than for MRBC-500 and MRBC-600, indicating that high temperature was more conducive to the generation of smaller mesopores, while low and medium temperatures were conducive to the formation of larger mesopores or large pores. The different pyrolysis time for the different pore size distributions were basically similar and without significant change patterns, indicating that the pyrolysis time had no significant effect on the evolution of the generation of MgO-modified biochar pore structure. The pore size region below 6 nm has a narrower distribution, while the medium and large pores above 6 nm have a wider distribution. As the MgCl_2_ impregnation concentration increased, the pore structure distribution of the modified biochar showed an increasing trend in the range of 8–140 nm, indicating that changing the impregnation concentration contributed to the generation of more mesopores and fewer macropores. This may be due to the fact that the increase of impregnation concentration increased the MgCl_2_ loading on the surface and pores of the biochar, and more pyrolysis reactions occurred on the surface of the biochar under high temperature, which resulted in the generation of rich pore structure.

### 3.5. Surface Morphology

**Figure 3:**
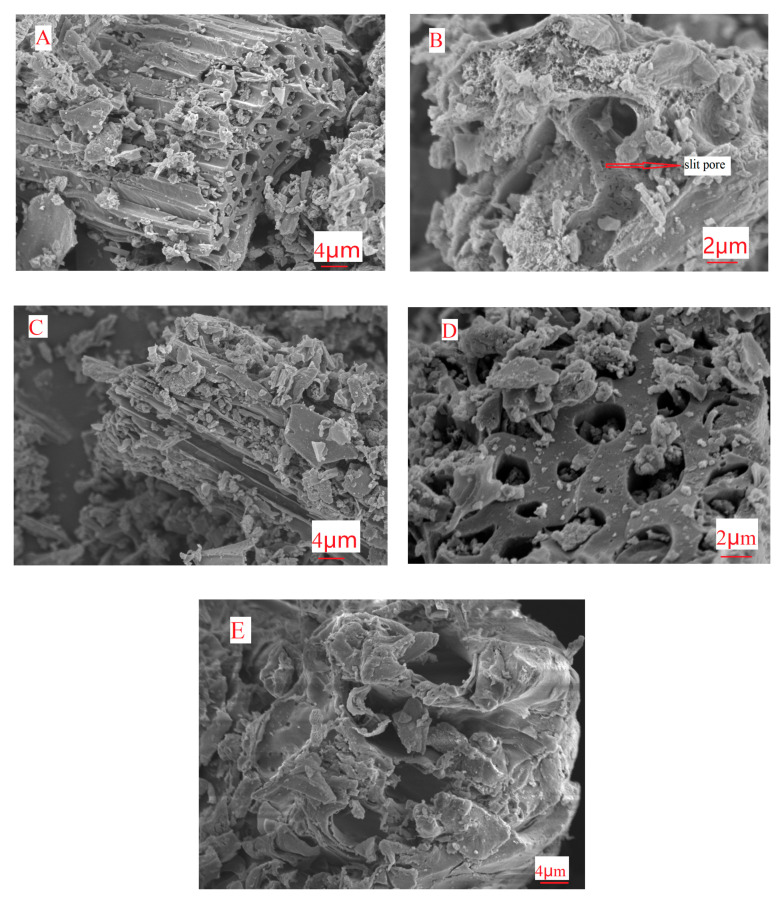
SEM Images of MRBCs (**A**–**D**) and RBC (**E**).

Scanning Electron Microscope (SEM) can be used to analyze the microstructures of biochar at the microscopic scale, which can directly reflect the surface morphology and structure characteristics of biochar. As a result that the surface structure of modified biochar is similar in general at different pyrolysis temperatures, holding time and impregnation concentration, only SEM images at different multiples were selected for analysis. It can be seen from the SEM scanning images (Figure 3) that there are pores of different pore sizes on the surface of the MRBCs, including slit pores and columnar pores, etc. In contrast, the surface of RBC-300 does not produce more pore structure. This conclusion was consistent with the nitrogen adsorption-desorption curve and the hysteretic loop type analysis results in the previous paper. In addition, the original skeleton structure of MRBCs can be maintained at higher pyrolysis temperatures. Furthermore, fine particulate matter can be found on the surface, cracks and pores of MRBCs, which may be the oxide of magnesium. 

### 3.6. Chemical Structure

**Figure 4:**
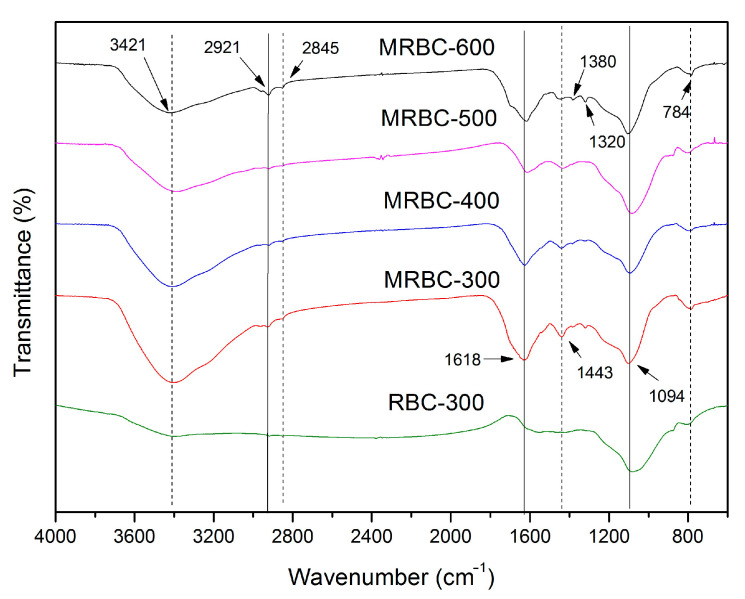
FTIR spectra of MRBCs and RBC.

Figure 4 shows Fourier transform infrared spectra of RBC and MRBCs. There were observable changes in surface functionality for the MgO-modified biochar materials produced under different pyrolysis temperature. With increasing carbonization temperature, the peak at 1443 cm^−1^ indicates that the bending vibration of the -COOH or -CHO gradually decreased [36]. Peaks representing C=C were observed at 1549–1607 cm^−1^ for all MRBCs. These were typical peaks observed for biochar, representing its aromatic structure [24]. The peak of 3421 cm^−1^ was attributed to the presence of hydroxyl group (-OH) stretching, and it represents a gradual decrease in the stretching vibration of the hydroxyl bond (-OH) as the carbonization temperature increases due to greater dehydration of the biochar materials [37]. The peak strength at 3421 cm^−1^ of MRBCs were enhanced when compared with RBC, indicating the O-H functional groups might be increased by MgO modification. The absorption peak at 1618 cm^−1^ was attributed to the stretching vibration of C=O and was present in almost all MRBCs. Moreover, the peak of MRBCs at 1618 cm^−1^ was stronger than that of RBC, indicating that the impregnation-modified biochar produced more C=O functional groups. The absorption peak of MRBCs at 1618 cm^−1^ showed a decreasing trend from 300 to 600 °C [38]. The peak of 2845 and 2921 cm^−1^ was associated with the presence of aliphatic group (-CHn) stretching [39], and the C-H stretching vibration absorption peak of modified biochar was higher than that of unmodified biochar. With increasing carbonization temperature, its absorption peaks increase at 2921 and 2845 cm^−1^, indicating that the MRBCs decompose and a more active aromatic structure was formed. When the temperature is increased from 300 to 500 °C, the biochar undergoes a violent pyrolysis reaction, producing products such as CO and H_2_, which eventually leads to a weakening of the absorption peaks at 2921 and 2845 cm^−1^ [40]. However, when the temperature is further increased by 600 °C, the biochar becomes more aromatic and more benzene ring structures are formed, leading to enhanced absorption peaks at 2921 and 2845 cm^−1^. MRBC has new absorption peaks at 1380 cm^−1^ and 1320 cm^−1^, which are C-O of phenols and CH_2_ symmetrical bending vibration. In addition, a new shoulder peak attributed to the C=O stretching vibration appears at 1700 cm^−1^ in MRBC-600, indicating that MRBCs were partially oxidized hydroxyl groups at 600 °C. Rice straw is rich in polymers such as lignin, hemicellulose and cellulose. The peak of the RBC-300 FITR spectrum at 1094 cm^−1^ is mainly due to their pyrolysis reaction. However, after biochar is modified by MgCl_2_ impregnation, as the pyrolysis temperature increases, intramolecular polycondensation occurs within the biochar, resulting in the production of volatile components. Therefore, C-O-C decreases with increasing temperature [41].

### 3.7. Crystal Structure

**Figure 5:**
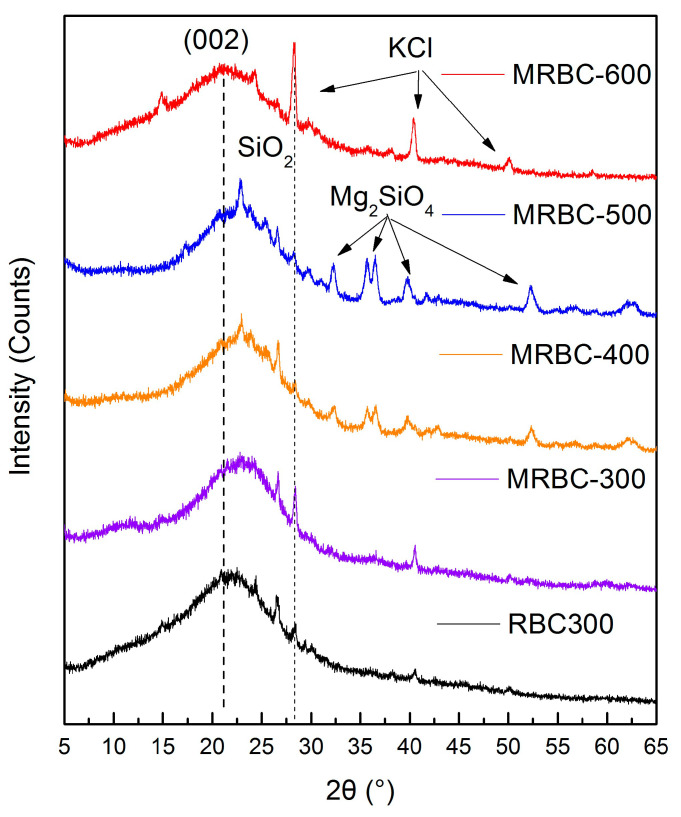
XRD patterns of RBC300 and MRBCs.

Figure 5 shows the XRD spectra of Mg-modified biochar and modified precursor biochar prepared at different temperatures. From Figure 5, it can be seen that the diffraction peak of the modified precursor biochar at 2θ = 20.9° belongs to the cellulose graphite microcrystalline d_002_ crystal plane. After the precursor biochar was modified by MgCl_2_, the intensity of the diffraction peak increased and moved to a high angle as the pyrolysis temperature increased up to 500 °C, indicating that the increase in temperature prompted the cellulose graphite microcrystals in the biochar to shrink the layer spacing and increase the superposition density, thus increasing the crystallinity and converting the chemical structure of the biochar into a more stable carbon compound [42]. The diffraction peak of inorganic crystalline SiO_2_ at 2θ = 28° was further found by X-ray diffraction standard card comparison. In addition, the characteristic diffraction peak of Mg_2_SiO_4_ (PDF#84-1402) appeared and gradually intensified in the modified biochar at 400 °C as the pyrolysis temperature increased, and disappeared when the pyrolysis temperature was 600 °C, which might be due to the chemical decomposition at high temperature. At the same time, the diffraction peak of KCl crystal (PDF#75-0296) appeared at 600 °C, which indicates that the increase in temperature led to the sintering and fusion of inorganic ions such as Si, K and Mg, resulting in inorganic minerals or alkali metals [43].

### 3.8. Elemental Analysis

**Figure 6:**
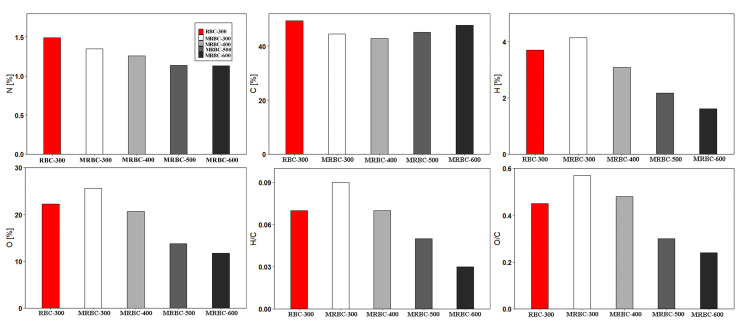
Elemental compositions of MRBCs and RBC.

Figure 6 shows the elementary compositions of MRBCs and RBC obtained from rice straw at carbonization temperatures of 300, 400, 500 and 600 °C. It is noteworthy that the trend in the elemental carbon contents decreased from 300 to 400 °C and increased from 400 to 600 °C. The phenomenon is due to the cellulose and hemicellulose reacted violently to produce volatile products at 300 to 400 °C, while the increase in the rate of carbonization as well as the development of aromatic carbon structures 400 to 600 °C. From Figure 6, it can be seen that the H, N and O contents of MRBC decreased with the increase of production temperature due to the loss of volatile components and dehydration of organic compounds as well as the cleavage of weak bonds within the feedstock structure [44]. The H, N and O contents decrease with increasing pyrolysis temperature with a range of 1.61–4.15%, 1.13–1.35% and 11.7–25.6%, respectively. Reduction in the oxygen content indicates the removal of carboxylic functional groups and the formation of aromatic ring structures with higher carbonization [45]. This is due to the dissipation of the functional groups containing hydrogen or oxygen [13]. In addition, the H/C and O/C ratio of biochar can reflect its aromaticity and polarity to some extent [46]. When the carbonization temperature was further increased to 600 °C, the hydrogen-carbon and oxygen-carbon atomic ratio both decreased, reflecting the enhanced aromaticity and weakened polarity of the biochar. This indicates the formation of aromatic compounds and a decrease in the content of polar compounds in the biochar at increased temperature [28]. Furthermore, the higher oxygen-carbon ratio in the MRBC surface might imply that the MRBC surface contains more abundant oxygen-containing functional groups, and thus the biochar exhibits higher hydrophilicity and polarity [47]. The trends of elemental composition and atomic ratios with increasing pyrolysis temperature were in accordance with the previous studies [48,49].

**Figure 7:**
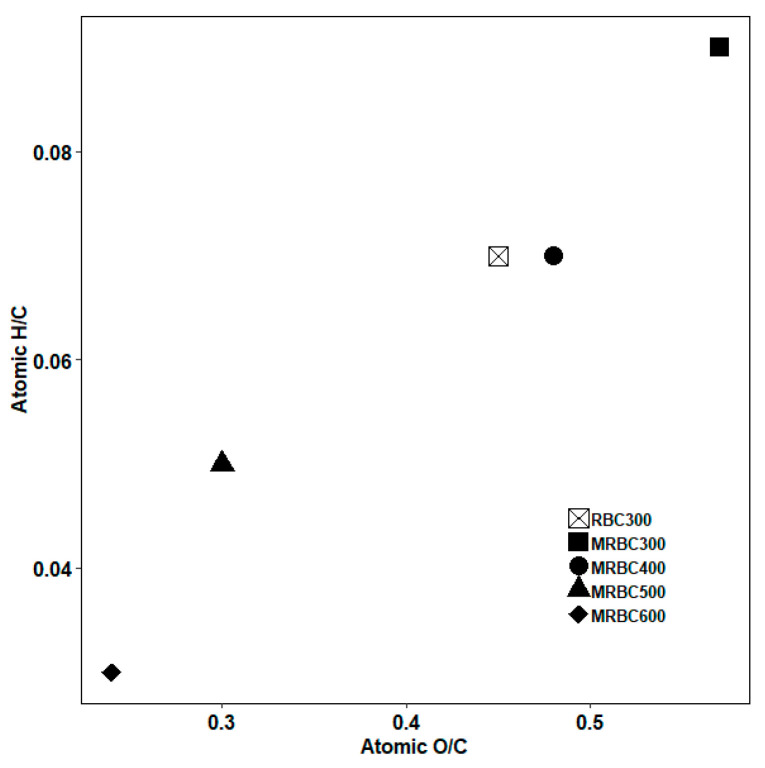
The Van Krevelen plot of elemental ratios for biochars produced at different pyrolytic temperatures.

Van Krevelen diagram is widely used to present the elemental content changes of biochar with pyrolysis temperature [48]. With the increase of pyrolysis temperature, the molar ratios of H/C and O/C decreased (Figure 7), while the decrease of H/C indicated that the carbonization degree of biochar increases.

## 4. Conclusions

The results of this study indicate that pyrolysis temperature was found to greatly influence on physicochemical properties of MRBCs while MgCl_2_ concentrations and pyrolysis time showed little effect on the specific surface area and pore structure of the modified biochar. With the increase of pyrolysis temperature, the total pore volume, microporous pore volume and mesopore volume of biochar increased. The pore structure was mainly dominated by smaller mesopores and the type of pore was slit pore. MRBC-2 h has the richest microporous structure and can be applied to the adsorption of benzene molecules etc. MRBC-2 m has the richest mesoporous structure and can be used for adsorption of colored dyes such as methylene blue, etc.

Peaks of MRBCs at 3421 cm^−1^, 1094 cm^−1^ and 1618 cm^−1^ decrease with increasing temperature suggesting the decrease in polar functional groups at high temperature. X-ray diffraction analysis showed that RBC-300 and MRBCs contained a large amount of graphitic microcrystalline cellulose carbon and different types of mineral salts, and temperature was a very important factor affecting biomass products. The H, N and O contents of MRBC decreased with the increase of production temperature due to the loss of volatile components and dehydration of organic compounds as well as the cleavage of weak bonds within the feedstock structure. Thus, MRBCs would be used as a potential functional material in wastewater treatment and soil remediation in the future.

## Figures and Tables

**Table 1:** Rice straw biochar prepared by different modification methods.

Samples	Modifier	Temperature (°C)	Pyrolysis Time (h)	Modifier Concentration (mol/L)
RBC-300	-	300	1	-
MRBC-300	MgCl_2_	300	1	1
MRBC-400	MgCl_2_	400	1	1
MRBC-500	MgCl_2_	500	1	1
MRBC-600	MgCl_2_	600	1	1
MRBC-0.5 m	MgCl_2_	500	1	0.5
MRBC-1 m	MgCl_2_	500	1	1
MRBC-1.5 m	MgCl_2_	500	1	1.5
MRBC-2 m	MgCl_2_	500	1	2
MRBC-0.5 h	MgCl_2_	500	0.5	1
MRBC-1 h	MgCl_2_	500	1	1
MRBC-2 h	MgCl_2_	500	2	1
MRBC-3 h	MgCl_2_	500	3	1

**Table 2:** Yield and pH of rice straw biochar (RBC) and MgO-modified biochars (MRBCs).

Samples	Yield (%)	pH
RBC-300	43.90 ± 0.40	9.5 ± 0.1
MRBC-300	87.80 ± 0.30	9.8 ± 0.0
MRBC-400	81.10 ± 0.50	10.0 ± 0.2
MRBC-500	66.95 ± 0.75	10.1 ± 0.1
MRBC-600	66.50 ± 1.50	10.2 ± 0.2
MRBC-0.5 m	68.43 ± 1.23	10.4 ± 0.1
MRBC-1 m	66.95 ± 1.35	10.1 ± 0.1
MRBC-1.5 m	65.48 ± 0.98	10.2 ± 0.0
MRBC-2 m	64.27 ± 1.17	10.2 ± 0.1
MRBC-0.5 h	67.15 ± 0.65	10.2 ± 0.0
MRBC-1 h	66.95 ± 1.85	10.1 ± 0.1
MRBC-2 h	67.15 ± 0.25	10.3 ± 0.1
MRBC-3 h	66.30 ± 1.30	10.4 ± 0.0

**Table 3:** Pore structure parameters of MRBCs and RBC.

Sample	Modified Temperature (°C)	Modifier	Specific Surface Area (m^2^/g)			Pore Volume (cm^3^/g)			Mesopore Porosity %	Average Pore Diameter (nm)
			S*_BET_*	S*_micro_*	S*_meso_*	V*_total_*	V*_micro_*	V*_meso_*		D
RBC-300	300	-	2.434	0.993	1.066	0.013	0.001	0.012	92%	20.977
MRBC-300	300	MgCl_2_	9.663	9.728	7.504	0.042	0.001	0.040	95%	17.210
MRBC-400	400	MgCl_2_	45.468	17.953	19.636	0.071	0.009	0.058	82%	6.257
MRBC-500	500	MgCl_2_	205.066	144.684	38.672	0.154	0.073	0.069	45%	2.999
MRBC-600	600	MgCl_2_	204.579	151.290	34.359	0.158	0.079	0.069	44%	3.094
MRBC-0.5 m	500	MgCl_2_	215.684	153.592	36.052	0.153	0.080	0.059	39%	2.837
MRBC-1 m	500	MgCl_2_	205.066	144.684	38.672	0.154	0.073	0.069	45%	2.999
MRBC-1.5 m	500	MgCl_2_	213.556	150.243	38.329	0.165	0.076	0.076	46%	3.082
MRBC-2 m	500	MgCl_2_	188.206	130.025	37.256	0.157	0.066	0.080	51%	3.347
MRBC-0.5 h	500	MgCl_2_	213.163	146.942	40.740	0.162	0.076	0.072	44%	3.033
MRBC-1 h	500	MgCl_2_	205.066	144.684	38.672	0.154	0.073	0.069	45%	2.999
MRBC-2 h	500	MgCl_2_	218.996	158.128	36.479	0.163	0.082	0.069	42%	2.980
MRBC-3 h	500	MgCl_2_	210.827	150.767	37.844	0.163	0.076	0.075	46%	3.096
KRSB [21]	400	FeCl_3_·6H_2_O	69.23	-	-	0.194	-	-	-	-
RSB [30]	500	AlCl3	147	-	-	0.062	-		-	-
BRP [31]	500	H_3_PO_4_	23.37							
